# Effect of Polyphenols From *Campomanesia adamantium* on Platelet Aggregation and Inhibition of Cyclooxygenases: Molecular Docking and *in Vitro* Analysis

**DOI:** 10.3389/fphar.2018.00617

**Published:** 2018-06-12

**Authors:** Caroline H. Lescano, Fernando Freitas de Lima, Camila B. Mendes-Silvério, Alberto F. O. Justo, Débora da Silva Baldivia, Cristiano P. Vieira, Eliana J. Sanjinez-Argandoña, Claudia A. L. Cardoso, Fabíola Z. Mónica, Ivan Pires de Oliveira

**Affiliations:** ^1^Department of Pharmacology, University of Campinas, Campinas, Brazil; ^2^Department of Chemical Engineering, University of Campinas, Campinas, Brazil; ^3^Faculty of Biological and Environmental Sciences, Federal University of Grande Dourados, Dourados, Brazil; ^4^Center for Natural Resource Studies, University of Mato Grosso do Sul, Dourados, Brazil; ^5^Department of Pharmacology, Institute of Biomedical Sciences, University of São Paulo, São Paulo, Brazil

**Keywords:** antioxidant, cyclic nucleotides, quercetin, thromboxane, COX inhibitors

## Abstract

*Campomanesia adamantium* is a medicinal plant of the Brazilian Cerrado. Different parts of its fruits are used in popular medicine to treat gastrointestinal disorders, rheumatism, urinary tract infections and inflammations. Despite its widespread use by the local population, the mechanisms involving platelet aggregation and the inhibition of cyclooxygenase by *C. adamantium* are unknown. This study evaluated the chemical composition, antioxidant activities and potential benefits of the *C. adamantium* peel extract (CAPE) and its components in the platelet aggregation induced by arachidonic acid in platelet-rich plasma. Aspects of the pharmacological mechanism were investigated as follows: platelet viability, calcium mobilization, levels of the cyclic nucleotides cAMP and cGMP, thromboxane B_2_ levels, and the inhibitory effects on COX-1 and COX-2 were studied *in vitro* and using molecular docking in the catalytic domain of these proteins. The major CAPE constituents standing out from the chemical analysis are the flavonoids, namely those of the flavones and chalcones class. The results showed that CAPE, quercetin and myricetin significantly decreased arachidonic acid-induced platelet aggregation; the assays showed that CAPE and quercetin decreased the mobilization of calcium and thromboxane B_2_ levels in platelets and increased cAMP and cGMP levels. Moreover, CAPE inhibited the activity of COX-1 and COX-2, highlighting that quercetin could potentially prevent the access of arachidonic acid more to the catalytic site of COX-1 than COX-2. These results highlight CAPE’s potential as a promising therapeutic candidate for the prevention and treatment of diseases associated with platelet aggregation.

## Introduction

Human platelets are anucleated cells that participate in many pathophysiological processes, including hemostasis and thrombosis (Ruggeri, 2002), clot retraction (Harrison, 2005), vessel constriction and repair, inflammatory diseases (Danese et al., 2004; Caudrillier et al., 2012), promotion of atherosclerosis (Ross, 1999), antimicrobial defense (Yeaman, 2010), cancer growth and metastasis (Gay and Felding-Habermann, 2011; Goubran et al., 2014), hepatic degeneration (Kirschbaum et al., 2017) and neurodegeneration (Jarre et al., 2014). Among their various roles, platelets manifest themselves more clearly in thrombosis (Xu et al., 2016). The driving force for haemostatic thrombus formation is a traumatic vascular injury. In response to this event, platelets undergo a highly regulated set of functional, but closely integrated responses involving adhesion, activation and aggregation (Ruggeri, 2002).

The platelet activation process begins with the binding of adhesive linkers and excitatory agonists to the cognate receptors on the platelet membrane and it is propagated by intracellular signaling reactions involving enzymes, substrates, and cofactors (Savage et al., 2001; Ruggeri, 2002). One of the main inducers of activation is TXA_2_, derived from the metabolism of AA, which is subsequently oxidized by cyclooxygenase (Davì and Patrono, 2007). When the activation is treated through thromboxane, the platelets are activated through receptors coupled to the G-protein, which convert an extracellular proteolytic cleavage into a signal (Savage et al., 2001), leading to the activation of phospholipase C by the hydrolysis of the phosphatidylinositol-4,5-diphosphate present in the membrane, producing second messengers, such as inositol-3-phosphate which, consequently, contribute to an increase of intracellular Ca^2+^ and the activation of enzymes dependent on this ion (Hourani and Cusack, 1991). When platelet aggregation occurs, there are physiological mechanisms to reverse excessive aggregation, such as the increase of the cellular synthesis of nitric oxide, which has been reported to produce both nucleotides, cyclic AMP and cyclic GMP, in platelets (Walter and Gambaryan, 2009).

*Campomanesia adamantium* (Myrtaceae genus) is a native fruit of the Cerrado used for nutrition. In popular medicine, the leaves and root are used for the treatment of diabetes and dyslipidemia (de Toledo Espindola et al., 2016) and the fruit is used to treat inflammation and rheumatism (Ferreira et al., 2013). Several studies have shown that different fruit species of the *Campomanesia* family have a biological effect, including antidiarrheal, antiproliferative (Lescano et al., 2016), antiulcerogenic (Markman et al., 2004), antiplatelet, antithrombotic, (Klafke et al., 2012), fibrinolytic and cardiovascular effects (Klafke et al., 2012). In this sense, this study sought to investigate the inhibitory mechanisms of *C. adamantium* peel extract (CAPE) in platelet aggregation, in addition to evaluating its effect on the mobilization of Ca^2+^ in platelets and the levels of cyclic nucleotides (cAMPc and cGMP) and TXB_2_. We also evaluated the antioxidant properties, chemical composition and inhibition of the COX-1 and COX-2 cyclooxygenase isoforms, revealing in detail how polyphenols should bind to the catalytic pocket of COX-1 and COX-2 with molecular docking. As such, we provide important pharmacological information that will aid in the interpretation of the medicinal effects associated with the extracts of *C. adamantium*.

## Materials and Methods

### Chemicals

MTT (3-(4,5-dimetylthiazol-2-yl)-2,5-diphenyltetrazolium bromide), DMSO (dimethyl sulfoxide), gallic acid, quercetin, xanthine, tannic acid, catechin, myricetin, Folin-Ciocalteau reagent, 6-hydroxy-2,5,7,8-tetramethyl-chroman-2-carboxylic acid (Trolox), 2,2-diphenyl-1-picrylhydrazyl (DPPH), 2,2′-Azino-bis 3-ethylbenzothiazoline-6-sulfonic acid (ABTS) and Fura 2-AM, were purchased from Sigma-Aldrich^®^ (MO, United States). Biochemical assay kits were from Cayman Chemical cAMP, cGMP, TXB_2_, and COX inhibitor screening. Solvents for chromatographic analyses (acetonitrile and methanol) were of LC–MS grade, obtained from J. T Baker (Pennsylvania, United States). Ultrapure water was generated by deionization (Millipore, Billerica, MA, United States).

### Plant Material and Preparation of the Extract

*Campomanesia adamantium* fruits were collected in regions of Cerrado biome located in the State of Mato Grosso do Sul, Brazil (22° 4′ 34.824″ S and 55° 8′ 33.936″ W), from Medicinal Plants Garden of Federal University of Grande Dourados (UFGD). A voucher specimen was deposited in the UFGD (n. 47620). Samples were pulped manually, and then pulp and peel were stored at -5°C until processing. Only peels were used for extract preparation, first samples were dehydrated at 40°C in a tray dryer (NG Scientific) with an air flow of 0.5 ms^-1^ for 72 h and triturated to a fine and uniform powder. Peels were extracted with absolute methanol for 21 days and filtrated. Extract was mixed, filtered, and concentrated under vacuum and lyophilized. The final powder was diluted in vehicle according to the experiment and then adjusted to the desired concentration to perform the tests.

### Phenolic Compounds

The concentration of phenolic compounds in the *C. adamantium* peel extract (CAPE) was determined according to Folin-Ciocalteu colorimetric method described by Shah et al. (2015). Briefly, 0.5 mL of the CAPE (10 mg/mL) was mixed with 2.5 mL of Folin-Ciocalteu reagent and 2 mL of sodium carbonate (Na_2_CO_3_) 14% (w/v). After 2 h the incubation at room temperature in the dark, absorbance was measured at 760 nm in a T70 UV/VIS spectrophotometer (PG Instruments Limited, Leicestershire, United Kingdom). A standard curve was prepared using gallic acid in the concentration range of 0.4–22.0 μg/mL. The total amount of phenolic compounds was expressed in milligrams of gallic acid equivalents (GAE) per gram of the CAPE. All experiments were performed in triplicate.

### Total Flavonoids

The concentration of total flavonoids in the CAPE was determined according to the method described by Chang et al. (2002). Briefly, 0.5 mL of the CAPE (10 mg/mL) was mixed with 0.1 mL of aluminum chloride (AlCl_3_.6H_2_O) at 10% (w/v) in absolute methanol, and 0.1 mL of sodium acetate 1 M and 2.8 mL of distilled water. The mixture was incubated for 40 min at room temperature, and the absorbance was read at 415 nm in a T70 UV/VIS spectrophotometer (PG Instruments Limited, United Kingdom). A standard curve was prepared using quercetin in the concentration range of 0.4–22.0 μg/mL. The total flavonoids were expressed in milligrams of quercetin equivalents (QE) per gram of the CAPE. All experiments were performed in triplicate.

### Condensed Tannins

The tannins condensed was determined through the colorimetric method described by Maxson and Rooney (1972) with modifications. The reaction mixture consisted in 1 mL of the CAPE with 4 mL vanillin-HCl (8% conc. aq. HCl and 4% vanillin in methanol). The mixture was incubated for 20 min at room temperature, and the absorbance was read at 500 nm in a T70 UV/VIS spectrophotometer (PG Instruments Limited, United Kingdom). A standard curve was prepared using catechin in the concentration range of 15–100 μg/mL. The condensed tannins concentration were expressed in milligrams of catechin equivalents (CE) per gram of the CAPE. All experiments were performed in triplicate.

### ABTS Analysis

The determinations of the ability to eliminate the ABTS free radicals were conducted according to the methodology previously described (Maria do Socorro et al., 2010), with modifications. The 2,2′-azino-bis(3-ethylbenzothiazoline-6-sulphonic acid) (ABTS) radical was prepared from a mixture of 5 mL of ABTS (7 mM) and 88 μL of potassium persulfate (145 mM) and incubated for 12–16 h at room temperature in the dark. After this period, the ABTS radical solution was diluted in absolute ethanol until an absorbance of 0.70 ± 0.02 at 734 nm was obtained in a T70 UV/VIS spectrophotometer (PG Instruments Limited, Leicestershire, United Kingdom). Reaction solutions, containing 30 μL of CAPE at different concentrations (5–100 μg/mL) was mixed with 3000 μL of the ABTS radical and the absorbance was taken at 734 nm after 6 min using a spectrophotometer. Ascorbic acid and 6-hydroxy-2,5,7,8-tetramethyl-chroman-2-carboxylic acid (Trolox) were used as positive controls. Two independent experiments were performed in triplicate. The percentage of inhibition of the ABTS radical was calculated according to the following equation, where Abs_control_ is the absorbance of ABTS radical without the tested sample: Inhibition of ABTS radical (%) = (1 - Abs_sample_/Abs_control_) × 100.

### DPPH Analysis

The DPPH radicals-scavenging activity of CAPE was determined according to the method previously proposed (Maria do Socorro et al., 2010), with modifications. A volume of 0.5 mL of CAPE at different concentrations (5–500 μg/mL) was added to 0.5 mL of DPPH solution (0.04 mM) and 1.5 mL of ethanol. The mixture was shaken and allowed to stand at room temperature for 10 min. Antioxidant activity was measured by recording the absorbance at 517 nm using a spectrophotometer (Varian Cary 50). Ascorbic acid was used as reference antioxidants. As a control, 0.5 mL of solvent used to dilute the CAPE was added to 0.5 mL of DPPH solution (0.04 mM) and 1.5 mL of ethanol. Two independent experiments were performed in triplicate. The percentage inhibition was calculated relative to the control using the following equation: Inhibition of DPPH radicals (%) = (1 - Abs_sample_/Abs_control_) × 100.

### Chromatographic Analysis

The CAPE in concentration of 20 μg/mL in methanol was analyzed using a High Performance Liquid Chromatograph Varian 210, with Diode Array Detector (DAD) and scan between 200 and 800 nm. The standard compounds used in HPLC analysis were previously isolated from extracts of leaves of *C. adamantium* (Cambess.) O. Berg (Coutinho et al., 2008). The standards were prepared in concentration of 1–10 μg/mL in methanol for calibration curve employed in the quantitative determination of compounds. It was used a reverse phase column C-18 (25 cm × 4.6 mm × 5 μm). Elution was performed in gradient system, with 40% methanol, 50% water and 10% acetonitrile taking 40 min to reach 80% methanol, 10% water and 10% acetonitrile, and 20 min to go back to the initial condition. The analysis time was 60 min, flow rate of 1 mL/min and injection volume of 20 μL.

### Human Platelet-Rich Plasma (PRP) Protocol Human

The study was approved by the Ethics Committee of State University of Campinas (n° 184.172). Written informed consent was obtained from every participant before blood donation. Human blood from healthy volunteers who had not received any medication within the previous 10 days was collected in sodium citrate 3.8% (1:9 v/v – one volume: nine volume of blood). The whole blood was centrifuged at room temperature (400 × *g*, for 12 min) to obtain the platelet-rich plasma (PRP), platelet-poor plasma (PPP) was prepared by further centrifugation at 800 × *g* for 12 min. Platelet aggregation was performed with an optical aggregometer (Profiler, 8 channel PAP-8 V2.0 optics. Bio-Data Corporation) at 37°C with 200 μL of PRP placed in glass cuvettes containing a disposable stir bar for constant stirring. Platelet aggregation was carried out in arachidonic acid (AA – 500 μM) stimulated platelets in the absence and in the presence of compounds.

### Platelet Viability Assay

The MTT assay was performed according to Mosmann (1983). Using 96-well plates, platelets (1.5 × 10^8^ platelets/mL) were incubated with CAPE (0.25, 0.5, 1, 2.5, 5, and 10 mg/mL) or quercetin or myricetin (10 μM) for 5, 15, 30, and 60 min. After the incubation period, platelets were incubated with 5 mg/mL of MTT solution for 3 h in a CO_2_ incubator. Then MTT dye was removed and 100 μL of solubilization solution (SDS 10% acidified) were added to the wells. Absorbance was measured at 540 nm using a microplate reader (Synergy^TM^ H1 Hybrid Reader, BioTek, United States). The positive control was 10% triton-X.

### Measurement of Intracellular Ca^2+^ Levels (Ca^2+^)_i_

Measurement of intracellular Ca^2+^ assay was carried out according to a previous study (Mendes-Silverio et al., 2012). Briefly, platelet-rich plasma obtained from ACD-C anti-coagulated (1:9 v/v – one volume: nine volume of blood) were added to washing buffer (140 mM NaCl, 0.5 mM KCl, 12 mM trisodium citrate, 10 mM glucose, 12.5 mM saccharose, pH 6.0) was incubated with the fluorogenic calcium-binding dye Fura-2 AM (1 μM) for 30 min at room temperature and again centrifuged at 800 *g* for 12 min. The pellet was gently resuspended in Krebs solution, and the number of platelets was adjusted to 1.5 × 10^8^ platelets/mL. Platelet aliquots of 300 μL were incubated with CAPE (0.25–10 mg/mL), quercetin and myricetin (10 μM) for 10 min. The samples were analyzed using Fluorometer (Synergy^TM^ H1 Hybrid Reader, Biotek, United States). For measurement total Ca^2+^ mobilization, the calcium concentration was adjusted to 1 mM with CaCl_2_. Following equilibration for at least 3 min, arachidonic acid (AA – 500 μM) was added to platelet suspension. To verify the Ca^2+^ mobilization from internal storage sites alone, EGTA (10 μM) was added to chelate the extracellular Ca^2+^. The fluorescence was monitored continuously with monochromator settings of 340 nm (excitation) and 510 nm (emission). The (Ca^2+^)_i_ levels was determined by applying the following equation: *Ca_i_^*2*+^= [Kd × (F - F*_min_*)]/(F*_max_ *- F)*, where *Kd =* 224, *F* is fluorescence of the sample, *F*_min_ is minimum fluorescence and *F*_max_ is maximum fluorescence.

### Extraction and Measurement of cAMP and cGMP

For extraction and measurement the cAMP and cGMP, platelet-rich plasma was pre-incubated with the phosphodiesterase inhibitor 3-isobutyl-l-methyl-xanthine (IBMX, 1 mM) for 20 min. Then, platelet-rich plasma (500 μL) was pre-incubated with CAPE (0.25, 2.5 e 10 mg/mL), quercetin or myricetin (10 μM) for 10 min, and stimulated with arachidonic acid (AA – 500 μM). After reaction, was addition of cold-acidified ethanol (67%, v/v) and samples were vigorously agitated for 30 s. Cell samples were centrifuged (4000 ×*g*, 30 min at 4°C). Supernatants were dried at 55–60°C under a stream of nitrogen.

### Extraction and Measurement of Thromboxane B_2_

For TXB_2_ measurement, first platelet-rich plasma was pre-incubated with CAPE (0.25, 2.5 and 10 mg/mL), quercetin or myricetin (10 μM) for 10 min, and stimulated with arachidonic acid (AA – 500 μM). The reaction was interrupted by the addition of cold EGTA (80 mM) and then centrifuged at 4000 ×*g* for 5 min (4°C), to obtain the supernatant which was stored at -20°C until the quantification. Preparation of tracer samples, standards and incubation with antibody were performed as described in commercially available kits (Cayman Chemical TXB_2_, Cyclic GMP or AMP EIA kit, Ann Arbor, MI, United States). The assays were performed in duplicates. The limit of detection is 1 pmol/mL for cGMP and 0.1 pmol/mL for cAMP and 0.5 pg/mL for TXB_2_.

### COX Inhibition

Inhibition of COX activity was determined by measuring the synthesis of PGE_2_ according to the instructions provided with the kit. Brief, COX-1 or COX-2 enzyme and heme were added to test tubes containing COX reaction buffer (total of 0.5 mL). The mixture was vortex mixed and exposed to vehicle (DPBS) or CAPE (10 mg/mL), quercetin or myricetin (10 μM) for 10 min at 37°C. This was followed by the addition of AA (100 μM) with further incubation for 2 min. Hydrochloric acid (1 M) was added to stop the COX reaction followed by chemical reduction with stannous chloride solution. A standard curve with PGE_2_ was generated at the same time and from the same plate and was used to quantify PGE_2_ levels produced in the presence of test compounds. Absorbance was read using a plate reader (Synergy^TM^ H1 Hybrid Reader, Biotek, United States) at 412 nm. Diclofenac (10 μM) was used as the reference compound, and all compounds were diluted in a buffer kit.

### Molecular Docking Simulations

The binding modes of quercetin with COX-1 and COX-2 enzymes were investigated using a molecular docking method. This compound was docked into catalytic site of cyclooxygenases using the DockThor program (de Magalhães et al., 2014). First, the three-dimensional structure of quercetin molecule was built and minimized with the ChemBioDraw Ultra 12.0 program. With the geometry optimized, the ligand was allocated into catalytic site using a DockThor platform adopting MMFF94 force field. The molecular docking was established in a cubic grid box (Δ*x*, Δ*y*, Δ*z* of 11, 11, 11 Å), centered at coordinate *x, y, z* 240, 114, 44 (for COX-1) and 22.90, 1.64, 32.52 (for COX-2) respectively, and discretization of the energy at 0.25 Å. The crystal structure of COX-1 complexed with Meloxicam and COX-2 complexed with Vioxx (5KIR) (Orlando and Malkowski, 2016) were obtained from the Protein Data Bank (PDB ID: 4O1Z) (Xu et al., 2014). We choose one of the subunits of the homodimer to investigate the ligand-protein interactions at the catalytic domain. The parameters are referred to as defaults in DockThor and the structures with positional root mean square deviation (rmsd) of up to 2 Å were clustered together. All results were visualized using VMD 1.9.2 (Visual Molecular Dynamics) program (Humphrey et al., 1996).

### Statistical Analysis

Data are shown as mean ± SEM and statistical significance was calculated using ANOVA followed by Tukey’s test assuming *P* < 0.05 as significant, calculated with GraphPad Prism (Version 6.0 - GraphPad Software, San Diego, CA, United States).

## Results

### Phenolic Compounds, Total Flavonoids, Condensed Tannins, and Antioxidant Activity

The concentrations of phenolic compounds, total flavonoids and condensed tannins in the CAPE were 138.09 mg GAE/g of extract, 77.10 ± 1.21 mg QE/g of extract and 3.64 ± 0.10 mg CE/g of extract, respectively. Tannins, a class of phenolic compounds, are subdivided into condensed tannins and hydrolysable tannins. This methodology quantified only condensed tannins, a group of important compounds due their antioxidant effect. In foods they influence taste and texture, providing foods with astringent characteristics due to their ability to precipitate proteins (Green, 1993; Rasmussen et al., 2005).

To investigate the antioxidant activity of the CAPE, we evaluated its ability to capture the free radicals ABTS and DPPH compared to the activities of known antioxidants, ascorbic acid and Trolox. The results of the half maximal inhibitory concentration of the free radicals ABTS and DPPH and the maximum activity presented by the CAPE are described in **Table 1**.

**Table 1:** IC_50_ and maximum activity of standard antioxidants and CAPE in the elimination of the free radicals ABTS and DPPH.

	Methods					

Sample	ABTS			DPPH		
	IC_50_ (μg/mL)	Maximum activity		IC_50_ (μg/mL)	Maximum activity	
		%	μg/mL		%	μg/mL
Ascorbic acid	18.51 ± 0.45	88.16 ± 0.56	50	10.09 ± 0.93	88.80 ± 0.23	20
Trolox	25.92 ± 1.42	82.22 ± 0.27	50	–	–	–
CAPE	68.38 ± 0.95	67.79 ± 1.57	100	163.70 ± 0.15	86.29 ± 0.59	500

*Values are shown as means ± SEM (n = 3).*

In the ABTS assay, the IC_50_ of the CAPE was 3.69 and 2.63 times larger than the ascorbic acid and Trolox controls, respectively. As for the DPPH assay, the IC_50_ found for CAPE was 16.22 times higher in relation to ascorbic acid. Although the values obtained for IC_50_ are very different for the two assays, the results suggest that the CAPE has antiradical potential and that the observed activity can be attributed to the concentration of phenolic compounds quantified in the extract.

### Identification of the Compounds in *Campomanesia adamantium* Peel

The extract was analyzed by HPLC-DAD in order to obtain the chromatographic profile of the samples and to allow for the identification and determination of the content of the compounds present in the *C. adamantium* peel extract (CAPE). The levels of the compounds present in the CAPE ranged from 39.17 to 155.16 mg/g (**Table 2**).

**Table 2:** Identified compounds and content of the methanolic extract from the *C. adamantium* peel by HPLC-DAD.

Peak	Compounds	Retention time (min)	Content (mg/g)
1	7-hydroxy-6-methyl-5-methoxyflavanone	18.01 ± 0.04	129.12 ± 0.19
2	5,7-dihydroxy-6-methylflavanone	24.06 ± 0.07	138.34 ± 0.21
3	5,7-dihydroxy-8-methylflavanone	24.89 ± 0.10	155.16 ± 0.23
4	5,7-dihydroxy-6,8-dimethylflavanone	26.83 ± 0.11	39.17 ± 0.10
5	2′,4′-dihydroxy-5′-methyl-6′-methoxychalcone	30.55 ± 0.11	54.12 ± 0.09
6	2′,4′-dihydroxy-3′,5′-dimethyl-6′-methoxychalcone	33.77 ± 0.16	53.04 ± 0.04

In study with extract ethanol:water (70:30) of the *C. adamantium* peel was identified 8 flavonoids (5,7-dihydroxy-6-methylflavanone, 5,7-dihydroxy-8-methylflavanone, 2′,4′dihyd roxy 6′-methoxychalcone, 7-hydroxy-5-methoxy-6-methyl flavavone, 5,7-dihydroxy-6,8-dimethylflavanone, quercetin, myricetin and 2′,4′-dihydroxy-3′,5′-dimethyl-6′-methoxy chalcone) (de Souza et al., 2017). The chemical composition identified partially corroborates with other studies that describe the chemical composition of the CAPE.

In the study performed by de Souza et al. (2017) on the extract ethanol:water (70:30) of the *C. adamantium* peel, evaluating anti-inflammatory activity and its chemical composition, the study presented flavonoids and chalcones as the extract main constituents, similar to found in our study. Furthermore, several phytoconstituents from different parts of this plant have been reported, such as quercetin, myricetin, myricitrin, gallic acid, ellagic acid, condensed tannins, among others (Ferreira et al., 2013; Campos et al., 2017; Viscardi et al., 2017). The presence of quercetin and myricetin in the extract of *C. adamantium* leaves has also been reported as the cause of their anti-inflammatory action (Ferreira et al., 2013).

### Anti-aggregating Effects of Extract and Its Constituents

**FIGURE 1:**
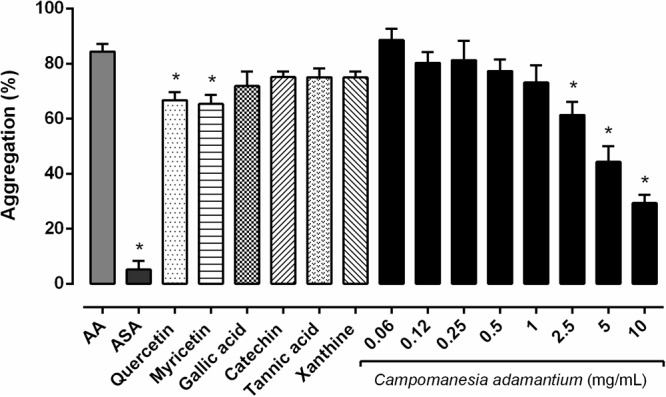
Inhibitory effect of the CAPE on human platelet aggregation *in vitro*. The platelets were stimulated with arachidonic acid (AA – 500 μM) after preincubation with the CAPE (0.06 – 10 mg/mL), acetylsalicylic acid (ASA – 100 μM), quercetin or myricetin (10 μM) for 10 min at 37°C. *n* = 5–6; One-way ANOVA followed by Tukey’s test; ^∗^*P* < 0.05 compared to arachidonic acid group.

It’s known that AA causes platelet aggregation mediated by the action of the hormone TXA_2_, which acts on the thromboxane receptors of the platelets. Considering that the CAPE has quercetin and myricetin in its composition, we examined whether the CAPE and some constituents inhibit AA-induced platelet aggregation at different concentrations. The antiplatelet effects of CAPE were evaluated using isolated human platelets against agonist-induced platelet aggregation, as shown in **Figure 1**. The extract showed a concentration-dependent inhibition of AA-induced platelet aggregation. A significant inhibition was obtained with 10 mg/mL 65.24 ± 3.04%, followed by 47.45 ± 5.63% in 5 mg/mL, 27.28 ± 4.77% in 2.5 mg/mL.

The extract typically has a mix of different chemical compounds, which can cause biological effects individually, or in synergism with other compounds (Gurib-Fakim, 2006). We analyzed six compounds (quercetin, myricetin, gallic acid, tannic acid, catechin and xanthine) present in our extract to investigate the possible effect on the platelets. Quercetin and myricetin showed inhibitory platelet activity on human platelets, 20.91 ± 2.87% and 22.54 ± 3.30%, respectively. Both are important compounds present in the CAPE, which have antiplatelet proprieties and may be acting synergistically in the platelet activities. According to previous reports, quercetin inhibited 50% of collagen-induced platelet aggregation. In addition, myricetin also attenuated 70% of the aggregation after AA stimulation (Tzeng et al., 1991).

### Platelet Viability

**FIGURE 2:**
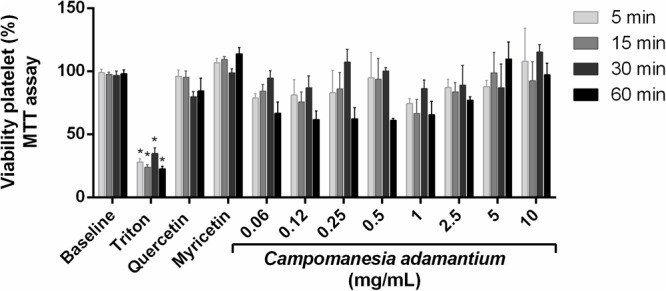
Effect of CAPE on platelet viability activity. Human platelets were treated with 10 mg/mL of extract, quercetin or myricetin (10 μM) for 5, 15, 30, and 60 min. Results are presented as mean + SEM. *n* = 9; Two-way ANOVA followed by Tukey’s test; ^∗^*P* < 0.05 compared to vehicle group.

The MTT assay was performed to assess whether the decrease in platelet aggregation could be due to platelet apoptosis. The results showed that CAPE at different concentrations during the times of 5, 15, 30, and 60 min did not decrease platelet viability (**Figure 2**). Some compounds present in the extract were analyzed, such as quercetin and myricetin the platelets showed good tolerance in the presence of the compounds. It is known that CAPE is well tolerated in T84 cells (colon carcinoma cells) (Lescano et al., 2016) and HepG2 cells (liver hepatocellular cells) (de Oliveira Fernandes et al., 2015).

### Decreased Mobilization of Intracellular Ca^2+^ by CAPE

**FIGURE 3:**
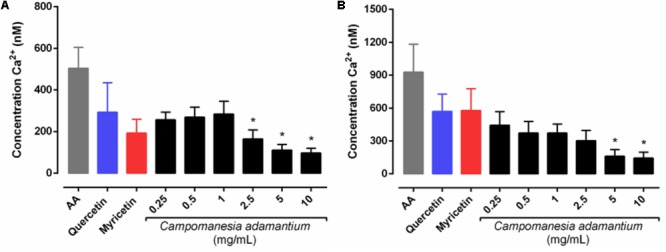
Effect of CAPE on intracellular Ca^2+^ levels in human isolated platelets activated with arachidonic acid (AA – 500 μM). Platelets (1.5 × 10^8^ cells/mL) loaded with Fura2-AM were pre-incubated with extract (0.25 – 10 mg/mL), quercetin or myricetin (10 μM). Assays were carried in Ca^2+^ free medium with EGTA to yield Ca^2+^ mobilization from internal storage sites **(A)** or out in the presence of Ca^2+^ to yield total influx of Ca^2+^ **(B)**. *n* = 5; One-way ANOVA followed by Tukey’s test; ^∗^*P* < 0.05 compared to arachidonic acid group.

The ability to measure calcium concentrations enables a clearer understanding of the role of other cellular messengers. Ca^2+^ mobilization was therefore studied in isolated human platelets activated with AA after pre-incubation with CAPE, quercetin or myricetin. The platelets stimulated with AA showed an increase in the mobilization of intra and extracellular Ca^2+^ (**Figures 3A,B**).

The experiments to observe the mobilization of calcium induced by AA (**Figure 3**) in the presence of CAPE and the compounds, produced data that shows that intracellular and total calcium mobilization decreased significantly at concentrations of 2.5–10 mg/mL and 5–10 mg/mL, respectively. This is in line with the results shown for the inhibition of platelet aggregation, since calcium (Ca^2+^) mobilization is a critical step in several aspects of platelet activation, such as aggregation, shape change and secretion (Kaibuchi et al., 1983).

### Increased Levels of Cyclic Nucleotides

**FIGURE 4:**
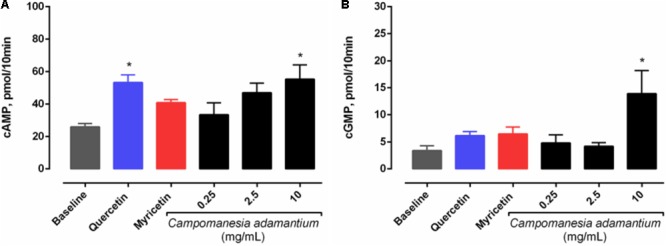
Effect of CAPE on **(A)** cAMP and **(B)** cGMP levels in platelets. Platelets were pre-incubated with extract (0.25, 2.5, and 10 mg/mL), quercetin or myricetin (10 μM) for 10 min, followed by activation with arachidonic acid (500 μM) for 5 min. Results are presented as mean + SEM, *n* = 3–6; One-way ANOVA followed by Tukey’s test; ^∗^*P* < 0.05 compared to baseline group.

The metabolic cascade of cyclic nucleotides is considered to be one of the most important pathways in platelet aggregation. In order to evaluate the possible intracellular signaling pathways involved in the inhibition by CAPE, we studied its effect on cyclic nucleotide levels. **Figure 4** shows that CAPE significantly increased cAMP (55.31 ± 8.76 pmol/10 min) and cGMP (19.84 ± 4.97 pmol/10 min) levels in platelets. In addition, quercetin was able to induce an increase in cAMP levels (53.35 ± 4.65 pmol/10 min).

### Decreased Levels of Thromboxane B_2_

**FIGURE 5:**
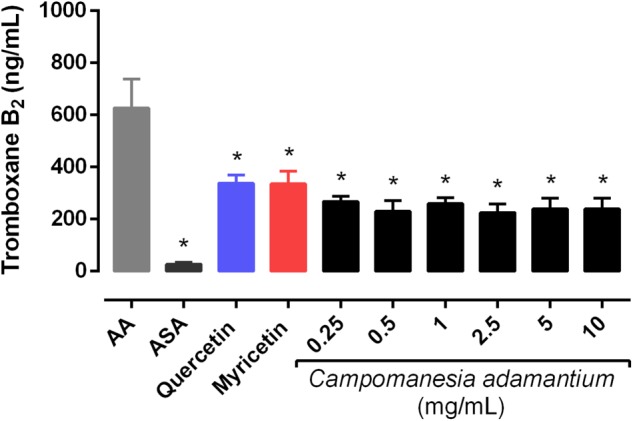
Effect of CAPE on platelet thromboxane B_2_ levels. Platelet-rich plasma was pre-incubated with extract (0.25 – 10 mg/mL), quercetin or myricetin (10 μM) for 10 min, followed by activation with arachidonic acid (AA – 500 μM). Results are presented as mean + SEM, *n* = 3–4; One-way ANOVA followed by Tukey’s test; ^∗^*P* < 0.05 compared to arachidonic acid group.

Arachidonic acid is a metabolite of TXA_2_ that acts as a potent mediator of platelet aggregation. For this reason, the effect of CAPE on the generation of the main metabolite of the COX pathway was analyzed. **Figure 5** shows that CAPE was able to inhibit it significantly at all concentrations. In addition, quercetin and myricetin decreased the TXB_2_ levels in the platelets by 54 ± 3.51% and 53 ± 4.85%, respectively.

### COX-1 and COX-2 Inhibition

Data presented in **Table 3** shows the results of the anti-inflammatory activity of the extract, quercetin and myricetin evaluated according to their ability to inhibit the COX-1 and COX-2 enzymes through an *in vitro* colorimetric COX (ovine) inhibitor assay. In summary, the CAPE and quercetin were able to significantly inhibit COX-1. In contrast, the inhibition of COX-2 was observed only for CAPE.

**Table 3:** Effects of the quercetin, myricetin and CAPE on COX-1 and COX-2 activity.

Compound	COX-1	COX-2
	
	Inhibition (%)	Inhibition (%)
Diclofenac (10 μM)	79.08 ± 6.93	86.54 ± 4.13
Quercetin (10 μM)	62.75 ± 14.73	15.43 ± 1.26
Myricetin (10 μM)	1.00 ± 0.65	10.35 ± 2.20
*C. adamantium* peel extract (10 mg/mL)	96.13 ± 0.75	89.88 ± 1.99

*Values are presented as mean ± SEM (n = 4).*

It is interesting to note that COX-1 is more constitutively expressed in platelets when compared to COX-2. In addition, its function is to convert AA to PGH_2_, followed by the metabolism of TXA_2_ and PGE_2_ (Moncada and Vane, 1978). We can assume from this result that CAPE and quercetin do not allow the AA to reach the catalytic domain of COX-1.

### Disposition of Quercetin at the Catalytic Site of COX-1 and COX-2

**FIGURE 6:**
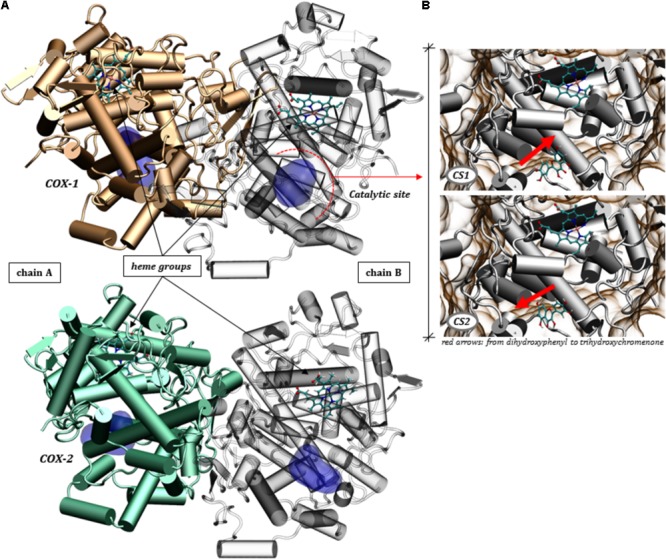
Three-dimensional structures of the COX-1 and COX-2 homodimers. **(A)** Highlight of the catalytic site domains and the heme grouping in both chains making up the proteins COX-1 (PDB ID: 4O1Z) and COX-2 (PDB ID: 5KIR). **(B)** The two conformational states (CS1 and CS2) assumed by quercetin in the molecular docking calculations for COX-1.

The biocatalytic reactions performed by COX-1 and COX-2 are conserved between isoforms, however, COX-2 can catalyze more efficiently a several substrates then COX-1, (this last more specific for AA) (Orlando and Malkowski, 2016). Both enzymes are composed of two peptide chains that form the homodimer (Smith et al., 2000) as shown in **Figure 6A**, with each subunit containing a catalytic site responsible for catalyzing the oxygenation of AA or analog substrates (Smith et al., 2011; Zou et al., 2012).

It’s known that the catalytic reaction is significantly affected by flavonoids (Bijak and Saluk-Bijak, 2017), which can act by inhibiting the conversion of AA to hydroperoxy endoperoxide, PGG_2_, by binding to the catalytic domain. In this sense, we sought to obtain a molecular view specifically of the interactions between quercetin and the residues of the catalytic site of COX-1 and COX-2 by docking simulations (see **Figure 6**).

The arrangement of quercetin in the catalytic site revealed itself in different ways, with two alternating conformations in the catalytic pocket of COX-1, shown in **Figure 6B**. These conformations are altered by a rotation of approximately 180 degrees, inverting the dihydroxyphenyl and trihydroxychromenone groups. This ability to accommodate the polyphenol at the catalytic domain must have a direct consequence on the ability to inhibit the catalysis of AA. It’s interesting to note that the protein-ligand affinities assumed by quercetin for these two conformations are very close, according to a docking score ranging from -8.96 to -8.90 for both conformational states (CS1 and CS2).

**FIGURE 7:**
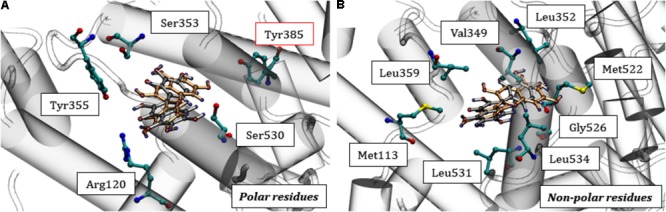
Interactions of quercetin at the catalytic site of COX-1. In **(A)** the polar residues are highlighted, mainly Tyr_385_ and in **(B)** the non-polar residues. Quercetin: orange (CS1) and gray (CS2) previously displayed in **Figure 6**.

The stabilization of quercetin in the catalytic pocket of COX-1 is due to the possibility of specific interactions with different residues, such as Met_113_, Ile_345_, Leu_359_, Leu_534_, Val_349_, Tyr_355_, Ser_353_, Leu_352_, Trp_387_, Phe_518_, Tyr_385_, Leu_535_, Met_522_, Leu_384_, Gly_526_, Ile_523_, Ala_527_, Pro_528_, Ser_530_, Leu_531_, Arg_120_, Leu_117_, and Val_116_. These residues and their neighbors are responsible for stabilizing ligands, as previously demonstrated by molecular modeling studies (Limongelli et al., 2010). In the catalytic domain, some residues are responsible for stabilizing quercetin according to the characteristics of its polar (**Figure 7A**) and non-polar (**Figure 7B**) side chains. The set of hydrophobic and polar interactions are responsible for stabilizing the polyphenol, especially through hydrogen bonds being that can be formed between the phenolic rings and the amino acid residues (Lescano et al., 2016).

**FIGURE 8:**
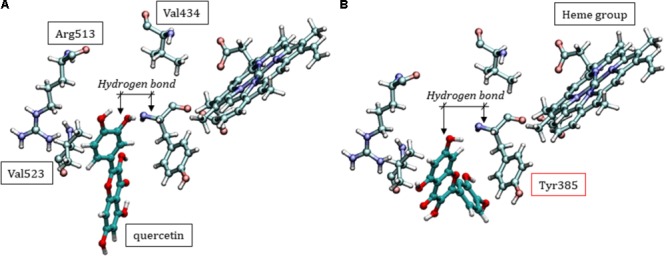
Two different conformational positions of quercetin in the catalytic site of COX-2, showing that inhibition can occur with similar interactions as those discussed for COX-1. **(A)** Specific residues in the COX-2 enzyme. **(B)** Hydrogen bond type interaction between Tyr_385_ and quercetin.

The molecular docking scores for COX-2 ranged from -7.136 to -8.811, suggesting a lower quercetin affinity with the catalytic site than COX-1, corroborating the *in vitro* analyses. It’s known that there are some differences in the catalytic domain of these proteins, e.g., substitutions of the residues Ile_434_, His_513_ and Ile_523_ (COX-1) by Val_434_, Arg_513_, and Val_523_ (COX-2), respectively, resulting in an increase of ¼ of the catalytic pocket volume (**Figure 8A**) (Garavito et al., 2002; Orlando and Malkowski, 2016). This property makes the protein more promiscuous, allowing it to catalyze a wider range of substrates and possibly influencing the lower affinity of quercetin for COX-2 compared with COX-1. Similar to COX-1, however, quercetin can accommodate itself within the catalytic pocket of COX-2. Then, interacting with Tyr_385_ residue and consequently affecting the oxygenation catalysis (**Figure 8B**).

## Discussion

This study evaluated the possible benefits of the peel extract of *C. adamantium* and its polyphenols in human platelets stimulated by the AA agonist. The medicinal plant *C. adamantium* is referred to as guavira, and traditionally used because of its anti-diabetic, anti-inflammatory (Coutinho et al., 2008), antidiarrheal (Lescano et al., 2016), antimicrobial, fibrinolytic and cardiovascular effects (Klafke et al., 2012). However, there is little data on the molecular mechanisms involved in the platelet aggregation effect. Through HPLC-DAD analysis, it was possible to discover some compounds present in the CAPE. They are derivatives of flavones and chalcones known as 5,7-dihydroxy-8-methylflavanone and 2′,4′-dihydroxy-5′-methyl-6′-methoxychalcone.

Furthermore, the phenolic compounds are considered natural antioxidants and a group of substances that is closely linked to the prevention of such diseases as cancer and atherosclerosis, with an anti-inflammatory, (Marinovic et al., 2015; Timmers et al., 2015), antiplatelet (Luzak et al., 2016) and vasodilator effects (Jofré et al., 2016). In addition to the phenolic acids and coumarins present in the CAPE, flavonoids and tannins are two important classes of phenolic compounds in the peel (Mann et al., 1994; Tuominen, 2013; Wu et al., 2013). The high concentration of phenolic compounds and flavonoids in the CAPE can explain its antioxidant capacity. This correlation is given by several factors, including the presence of different active compounds that are able to act as antioxidant agents and the synergistic effects of different compounds.

The results obtained from the ABTS and DPPH assays suggest that the flavonoids identified in CAPE (de Souza et al., 2017) may contribute to the elimination of free radicals and to the effects described in this study. Consequently, several studies have correlated the content of polyphenols and flavonoids with the antiradical effect of plant extracts (Kumar et al., 2010; Santana et al., 2012; Santos et al., 2016). The antioxidant capacity of flavonoids occurs through the donation of hydrogen atoms from an aromatic hydroxyl group to the free radical, leading to the stabilization of the free radical and consequently the prevention of diseases (Duthie et al., 2003). The search for natural antioxidants is therefore of substantial interest to human health, since flavonoids, for example, are reported to inhibit platelet aggregation (Assefa et al., 2016).

Since CAPE has anti-inflammatory effects and anti-oxidative activities (de Souza et al., 2017), we explored its effect on platelet aggregation and the potential mechanisms underlying this effect. In our study, we sought to evaluate the effects of CAPE, quercetin and myricetin were selected as representative phenolic compounds, since they are present in the extract and probably contribute to its inhibitory activity in platelet aggregation. In addition, CAPE, quercetin and myricetin reduced the aggregation of platelets stimulated by the AA agonist, without decreasing platelet viability. These findings led us to explore other underlying effects and mechanisms of CAPE in platelet aggregation. Because platelets participate in the development of two of the major health problems involving cardiovascular disease, thrombosis and atherosclerosis (Davì and Patrono, 2007). Both are among the leading causes of morbidity and mortality worldwide and their impact will increase further in the coming decades (Thom et al., 2006). It’s also important to highlight the use of antithrombotic therapy, considered as one of the pillars in the management of individuals at risk of thrombosis, as well as in patients with a history of coronary events (Zusman et al., 1999).

From a mechanistic point of view, CAPE flavonoids can exert their protective effects by inhibiting COX-1, attenuating the inflammatory pathways with additional effects on the second signaling pathways of cAMP and cGMP. This way, CAPE polyphenols can exert their effects, acting through different mechanisms in addition to their antioxidant capacity, as recently revised (Faggio et al., 2017).

The cAMP/PKA and cGMP/PKG pathway plays an essential role in the signal transduction by cyclic nucleotides associated with platelet activation and aggregation. PKA is a member of the kinase protein family, which transduces signals from the second cAMP messenger, and similarly activates cGMP-activated PKG (Francis et al., 2010). The phosphorylation of both promotes inhibition of platelet aggregation, since it is described that VASP phosphorylation occurs in response to the regulation by cyclic nucleotides (i.e., elevation of cAMP and cGMP levels), which is closely correlated with platelet inhibition and, in particular, with the inhibition of the fibrinogen bond to the integrin of human αIIbβ3 platelets (Aszódi et al., 1999).

However, several studies have shown the potential therapeutic effect of phenolic compounds, including quercetin, through the modulation of platelet aggregation against cardiovascular diseases (Pignatelli et al., 2000; Hubbard et al., 2003). Several action mechanisms have been reported, including inhibition of the AA pathway, suppression of cytoplasmic Ca^2+^ increase (Faggio et al., 2017) and inhibition of thromboxane formation (Guerrero et al., 2005). Here, CAPE and quercetin reduced platelet calcium mobilization. This ion regulates many events that lead to platelet aggregation, such as the activation of phospholipase A_2_, which hydrolyzes membrane phospholipids, leading to the production of AA and then thromboxane synthesis (Moncada and Vane, 1978).

Interestingly, elevated TXA_2_ levels in platelets upon AA activation decreased significantly in the presence of CAPE. In line with the observed results, CAPE was able to inhibit the COX-1 enzyme *in vitro*. Our results indicate that quercetin may act in a similar way as ASA in inhibiting COX-1, which explains the decrease in the TXA_2_ production. These results are consistent with previous reports on the anti-inflammatory effect of *C. adamantium* extract *in vitro, in vivo* (Ferreira et al., 2013; Lescano et al., 2016; de Souza et al., 2017) and the immunomodulatory use of quercetin (Sternberg et al., 2008).

Given the importance of the polyphenols in *C. adamantium*, especially quercetin, we observed that the molecular mechanisms involved in the inhibition of COX-1 by this compound can have two origins: (1) the chemical modification of important residues for enzymatic catalysis; (2) quercetin deposition in the catalytic domain and steric hindrance of AA entry. In the first hypothesis, the mechanism may be similar to that observed for ASA, where acetylation of the Ser_530_ residue inactivates COX-1, preventing bioconversion of AA to PGG_2_ (Bijak and Saluk-Bijak, 2017; Zacharias-Millward et al., 2017). In the case of quercetin, some alternative reaction to acylation could happen. However, the most consistent hypothesis must be based on the persistence of quercetin in the catalytic pocket without chemically altering the protein residues, only being maintained in the domain by intermolecular interactions. Such permanence would impede the entry of AA, with consequent catalytic inhibition of COX-1 and no possibility of PGG_2_ formation.

In a special way, Tyr_385_ participates directly in the mechanism of AA catalysis, transferring an electron to the heme group and, consequently, generating a tyrosyl radical in the catalytic site of COX, initiating the cyclooxygenase reaction (Rouzer and Marnett, 2009). It is interesting to observe the proximity of quercetin with the residue Tyr_385_, which should have the consequence preventing the participation of this residue in the reactive mechanism for obtaining PGG_2_. It is clear that quercetin can act by preventing AA from entering the catalytic site, but it may also form hydrogen bonds with the Tyr_385_ side chain. Quercetin could therefore act directly on the catalysis mechanism: (i) interacting with Tyr_385_ and (ii) preventing the entrance of the substrate.

## Conclusion

In conclusion, this study revealed for the first time that CAPE exerts antiplatelet activity via COX-1 inhibition and thus decreases platelet aggregation, increasing cyclic nucleotide levels, decreasing intracellular and total calcium mobilization and TXB_2_ levels. Additionally, we showed that the compound quercetin present in the extract was able to inhibit the access of AA to the catalytic site of COX-1. The CAPE and phenolic compounds, such as quercetin, therefore show therapeutic potential for use in the prevention and treatment of diseases associated with platelet aggregation.

## Author Contributions

CL, FFdL, CM-S, AJ, DdSB, CV, ES-A, CC, FM, and IPdO contributed to the literature database search, data collection, data extraction, data analysis, and writing of the manuscript. CL, FFdL, CC, and IPdO performed data analysis and rationalization of the results. All authors read and approved the final version of the manuscript.

## Conflict of Interest Statement

The authors declare that the research was conducted in the absence of any commercial or financial relationships that could be construed as a potential conflict of interest.

## Abbreviations

AA: arachidonic acid
ANOVA: analysis of variance
ASA: acetylsalicylic acid
cAMP: cyclic adenosine monophosphate
CAPE: *Campomanesia adamantium* peel extract
cGMP: cyclic guanosine monophosphate
COX-1: cyclooxygenase 1
COX-2: cyclooxygenase 2
HPLC: high performance liquid chromatography
PGE_2_: prostaglandin-endoperoxide synthase 2
PGG_2_: prostaglandin G_2_
PGH_2_: prostaglandin H_2_
PKA: protein kinase A
PKG: protein kinase G
TXA_2_: thromboxane A_2_
TXB_2_: thromboxane B_2_
VASP: vasodilator-stimulated phosphoprotein
